# Role of GABAB Receptor and L-Arg in GABA-Induced Vasorelaxation in Non-diabetic and Streptozotocin-Induced Diabetic Rat Vessels

**DOI:** 10.6091/ibj.1461.2015

**Published:** 2015-04

**Authors:** Fatemah Kharazmi, Nepton Soltani, Sana Rezaei, Mansoor Keshavarz, Leila Farsi

**Affiliations:** 1 *Dept. of Physiology, Faculty of Medicine, Molecular Medicine Research Center, Hormozgan University of Medical Science, Bandar Abbas, Iran; *; 2 *Dept. of Physiology, Faculty of Medicine, Tehran University of Medical Science, Tehran, Iran*

**Keywords:** Gamma amino butyric acid (GABA), Diabetes, GABA_B_ receptor

## Abstract

**Background:**

Hypertension is considered an independent risk factor for cardiovascular mortality in diabetic patients. The present study was designed to determine the role of gamma amino butyric acid B (GABA_B_) receptor and L-arginine (L-Arg) in GABA-induced vasorelaxation in normal and streptozotocin-induced diabetic rat vessels.

**Methods:**

Diabetes was induced by a single i.p. injection of streptozotocin (STZ, 60 mg/kg). Eight weeks later, superior mesenteric arteries of all groups were isolated and perfused according to the McGregor method.

**Results:**

Baseline perfusion pressure of STZ diabetic rats was significantly higher than non-diabetic rats in both intact and denuded endothelium. In the presence of faclofen, a selective GABA_B_ receptor blocker, GABA-induced relaxation in intact and denuded endothelium mesenteric beds of STZ diabetic rats was suppressed, but this response in non-diabetic rats was not suppressed. Our results showed that in the presence of L-Arg, a nitric oxide precursor, GABA induced vasorelaxation in both diabetic and non-diabetic vessels.

**Conclusion:**

From the results of this study, it may be concluded that the vasorelaxatory effect of GABA in diabetic vessel is mediated by the GABA_B_ receptor and nitric oxide, but it seems that in non-diabetic vessel GABA_B_ receptor does not play any role in GABA-induced vasorelaxation, but nitric oxide induced GABA relaxation in non-diabetic vessel.

## INTRODUCTION

Diabetes mellitus is one of the most common chronic global diseases in the world [1]. The major types of diabetes mellitus are type 1 and type 2. Sustained hyperglycemia is a common result of uncontrolled diabetes that could disturb the functions of organs, such as heart, eyes, kidneys, and nerves system, mainly through deteriorating blood vessels supplying the organs [1]. Accordingly, vascular complications are important causes of morbidity and mortality in diabetic patients, and hypertension is also considered as an independent risk factor for cardiovascular mortality in such patients [2]. On the other side, it has been reported that gamma amino butyric acid (GABA) is decreased in diabetic patients [3, 4]. It is well known that GABA is the major inhibitory neurotransmitter that is found in the brain and the pancreas [3, 5], and the inhibitory signaling of GABA is mediated by GABA_A_, GABA_B_, and GABA_C_ receptors [6]. 

Our previous study showed that GABA replacement therapy preserves β-cell mass in severe diabetic mice and prevents development of type I diabetes, and in severely diabetic mice, GABA restores β-cell mass and reverses the disease [7]. We also showed that GABA not only can induce endothelium vasorelaxation in control and streptozotocin (STZ) diabetic groups but also had a different effect on intact and denuded endothelium [8]. Our previous finding supports the hypothesis that the GABA relaxatory effect is mediated by the GABA_A_ receptor and nitric oxide system in both STZ diabetic and non-diabetic vessels [9]. However, regarding to our finding, there are still remained two questions. First, does GABA_B_ receptor play a role in GABA-induced vasorelaxation in diabetic and non-diabetic vessels? Second, will GABA-induced vasorelaxation raise vessel nitric oxide level? Although some researchers have found that GABA-induced vasorelaxation is not suppressed by GABA_B_ receptor antagonists in normal vessels [10], there has been limited research on the impact of the GABA mechanism on vascular diabetics.

Therefore, the present study was designed to determine the role of GABA_B_ receptor and L-arginine (L-Arg) in GABA-induced vasorelaxation in normal and STZ-induced diabetic rat vessels.

## MATERIALS AND METHODS

***Animals.*** The animals were handled in accordance with the criteria outlined in the Guide for Care and Use of Laboratory Animals (NIH US publication 86-23 revised 1985). Locally produced male Wistar rats (body weight 180–250 g) were used. All animals were maintained at a constant temperature (22 ± 0.5oC) with a fixed 12:12-h light/dark cycle. Animals were divided into 11 groups: six STZ diabetics and five non-diabetics (n = 6 in each group). Eight weeks later, all animals were anesthetized by i.p. injection of ketamine HCl (50 mg/kg) and mesenteric vascular beds were prepared as originally described by McGregor [11].

***Diabetes induction.*** Diabetes was induced with a single i.p. injection of STZ, 60 mg/kg. Before intervention and ten days and eight weeks after STZ injection, non-fasting blood glucose and insulin levels were determined using a glucometer (Ascensia ELITE XL glucometer, Germany) and insulin ELISA kit (Crystal chem., Chicago, USA), respectively. Rats with blood glucose levels of 300 mg/dl or above were considered to be diabetic.

***Preparation of mesenteric vascular bed.*** In brief, the abdominal wall was opened, the superior mesenteric artery was exposed and cannulated, and gently flushed with modified Krebs-Henseleit solution (containing [mM]: NaCl, 118; KCl, 4.7; CaCl2, 2.5; MgSO4, 1.2; glucose, 2; NaHCO3, 2.5; NaHPO4, 1.2) concomitantly bubbled with a mixture of 95% O2 and 5% CO2 (final pH 7.4), and warmed to 37oC. The mesentery was isolated from the intestine and placed in a water-jacked perfusion chamber maintained at 37oC. The preparation was perfused at 1 ml/min with modified Krebs-Henseleit solution (containing [mM] NaCl, 118; KCl, 4.7; CaCl2, 2.5; MgSO4, 1.2; glucose, 2; NaHCO3, 2.5; NaHPO4, 1.2) by a peristaltic pump (Meredos GmbH, Germany). The tissue was prevented from drying by superfusion with 0.1 ml/min modified Krebs-Henseleit solution. Perfusion pressure was monitored via a T tube inserted between the pump and the inflow cannula. This cannula was connected to a pressure transducer (MLT0380 ADInstruments, Australia), and the recording was performed by Power Lab System (16SP, ADInstruments). After a 30-minute equilibr-ation, the vascular bed was constricted by Krebs-Henseleit solution containing phenylephrine, an α1-adrenoceptor agonist (1 mM for intact and denuded diabetic groups and 3 mM for intact and denuded control groups), to induce 70-75% of maximal vasoconstriction (the doses of phenylephrine were chosen according to the phenylephrine dose response curve, data not shown in the results) and then allowed to reach a plateau and stabilize. Increasing concentrations of GABA of 1, 10, 20, and 50 µM were added to the medium every 15 minutes and the perfusion pressure was recorded. 

***Endothelial denudation.*** For achievement of endothelial denudation, the preparation was perfused with distilled water for five minutes [12].

***Nitric oxide production.*** For production of nitric oxide, L-Arg, a nitric oxide precursor, at a dose of 0.01 M was added to the medium 20 min before phenylephrine administration. Then the phenylephrine concentration was adjusted to achieve 70-75% of the maximum contractile response.

***GABAB and GABAA receptor inhibition.*** For inhibition of GABAB and GABAA receptors, faclofen, a selective GABAB receptor and bicuculline, a selective GABAA receptor blocker, at doses of 25 μM and 0.1 mM, respectively were added to the medium 20 min before phenylephrine administration. Then the phenylephrine concentration was adjusted to achieve 70-75% of the maximum contractile response.

***Drugs.*** The following drugs were used in this study. STZ was purchased from Sigma (USA) and dissolved in 1 ml normal saline immediately before use. GABA, L-Arg, biculline, phenylephrine, and faclofen were obtained from Sigma (St. Louis, MO, USA). Ketamine HCl was obtained from Rotexmedica (Trittau, Germany). 

***Statistical analysis.*** Data were expressed as mean ± S.E.M. Comparisons between groups were analyzed by student’s t-test, and two-way analysis of variance, followed by Tukey’s test using SPSS software version 20. P < 0.05 was considered significant. 

## RESULTS

No significant differences were found between the groups before the intervention. Ten days after STZ injection, plasma glucose levels in diabetic group were significantly increased from 107.5 ± 6.9 mg/dl to 402.2 ± 58.9 mg/dl. Also, eight weeks after diabetes induction, plasma glucose levels remained significantly elevated (Table 1).

**Table 1:** Plasma glucose concentrations before and eight weeks after diabetes induction

**Group ** **(n = 30 each)**	**Glucose (mg/dl)**	
	**Before intervention**	**Eight weeks after STZ or saline injection**
Non-diabetic control	105.64 ± 7.35	112.67 ± 4.1
Diabetic	105.70 ± 6.90	443.50 ± 4.1*

* significant difference between diabetic and non-diabetic control groups eight weeks after STZ or saline injection (*P* < 0.0001). Data are expressed as mean ± SEM.); STZ, streptozotocin

**Fig. 1:**
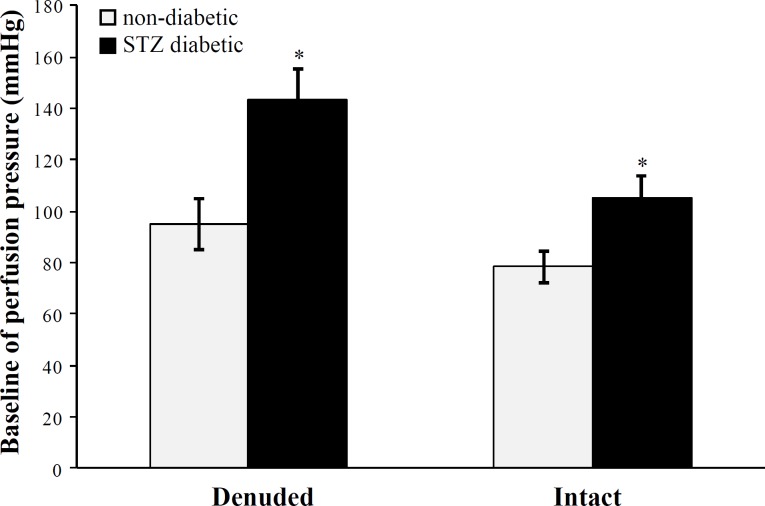
Baseline perfusion pressure (mmHg) of mesenteric vascular beds in non-diabetic and STZ diabetic groups with intact and denuded endothelium (six rats in each group). Data were expressed as mean ± SEM. ^*^Significant difference between STZ diabetic and non-diabetic groups with intact and denuded endothelium (*P* < 0.001); STZ, streptozotocin

***Mesenteric bed response.*** Baseline perfusion pressure in the STZ diabetic group was significantly (P < 0.001) higher than non-diabetic rats in both intact and denuded endothelium (Fig. 1).

**Fig. 2:**
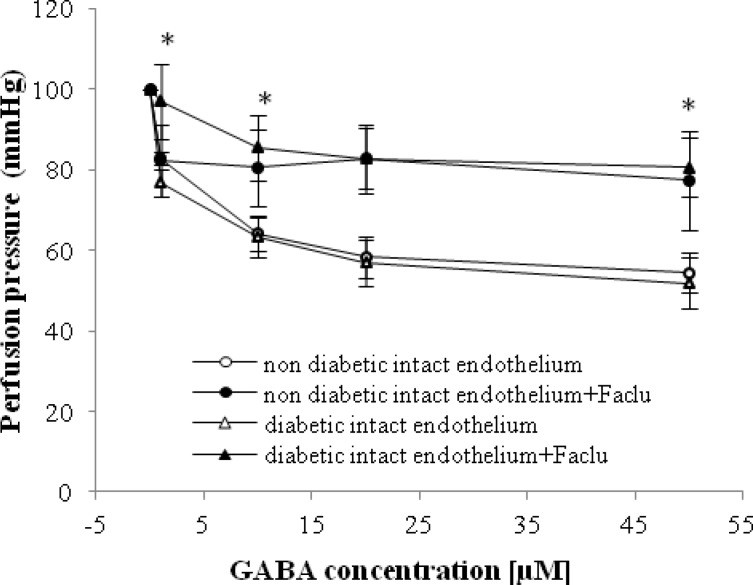
Dose response curves of gamma amino butyric acid (GABA) in mesenteric vascular beds of non-diabetic intact endothelium, non-diabetic intact endothelium + Faclofen (Faclu), diabetic intact endothelium, and diabetic intact endothelium + Faclu groups (six rats in each group). Data were expressed as mean ± SEM. ^*^Significant difference diabetic intact endothelium and diabetic intact endothelium + Faclu groups (*P* < 0.0001).

GABA at doses of 1-50 μM significantly decreased the perfusion pressure in diabetic and non-diabetic groups with intact endothelium in a dose-dependent manner (Fig. 2). The GABA-induced vasorelaxation in both diabetic and non-diabetic groups started with a dose of 1 µM of GABA, and it reached steady state at a dose of 20 µM (Fig. 2). 

In the presence of faclofen (0.1 mM), GABA-induced relaxation in intact endothelium mesenteric beds of diabetic rats was suppressed, and perfusion pressure in this group did not change (Fig. 2). Significant differences were observed at GABA concentrations of 1 to 50 µM in the presence and absence of faclofen in diabetic group with intact endothelium (Fig. 2). However, GABA-induced vasorelaxation in non-diabetic group was not suppressed in presence of faclofen. 

**Fig. 3:**
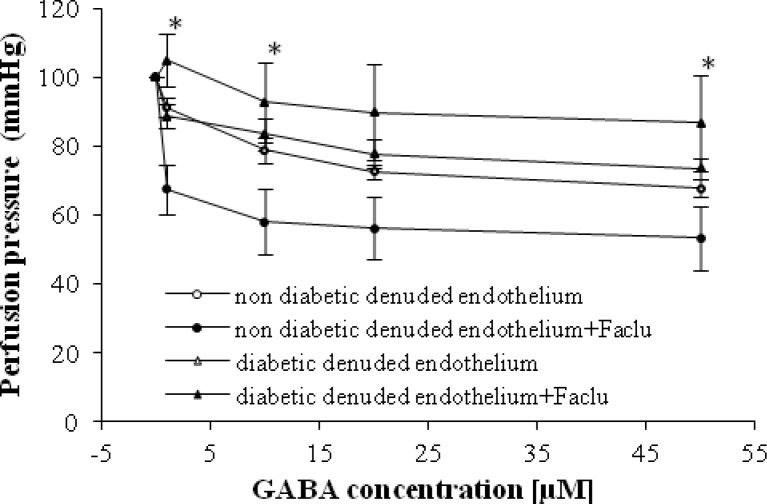
Dose response curves of gamma amino butyric acid (GABA) in mesenteric vascular beds of non-diabetic denuded endothelium, non-diabetic denuded endothelium + Faclofen (Faclu), diabetic denuded endothelium, and denuded endothelium + Faclu groups (six rats in each group). Data were expressed as mean ± SEM. ^*^Significant difference between non-diabetic denuded endothelium and non-diabetic denuded endothelium + Faclu (*P* < 0.0001).

In the present study, we showed that in the presence of faclofen (0.1 mM), GABA-induced relaxation in the denuded endothelium mesenteric beds of diabetic rats was suppressed and perfusion pressure did not change (Fig. 3). Significant differences were observed at GABA concentrations of 1 µM in the presence and absence of faclofen in diabetic group with denuded endothelium (Fig. 3). It is interesting that we found significant relaxation in non-diabetic group with denuded endothelium in presence of faclofen (Fig. 3).

**Fig. 4:**
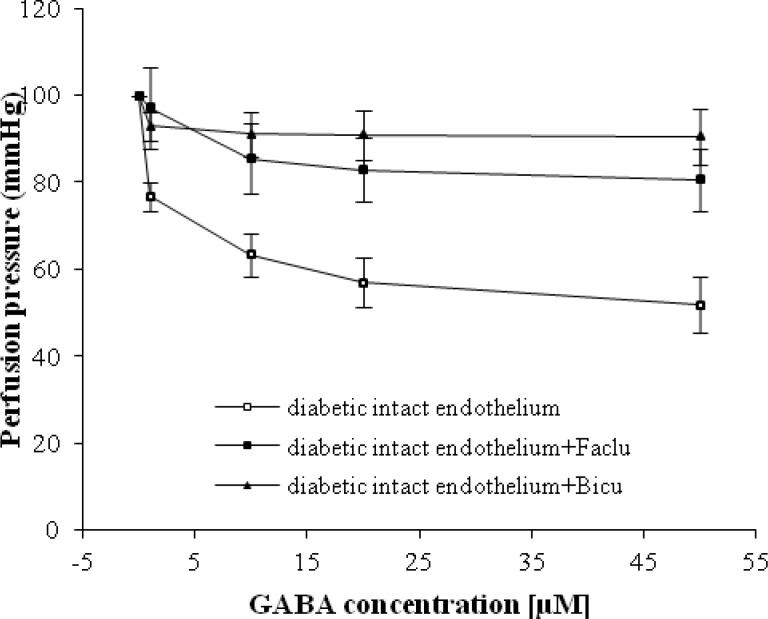
Dose response curve of gamma amino butyric acid (GABA) in mesenteric vascular bed with intact endothelium in diabetic, diabetic intact endothelium + Faclofen (Faclu), and diabetic intact endothelium + Biciculline (Bicu) (six rats in each group). Data were expressed as mean ± SEM.

Our results in Figure 4 showed that there is no significant difference between dose-response curve of GABA in mesenteric vascular bed with intact endothelium in diabetic rats in the presence of bicuculline and faclofen. There was also no significant difference between the slopes of percentage response curves in diabetic group with intact in presence of bicuculline and faclofen.

**Fig. 5:**
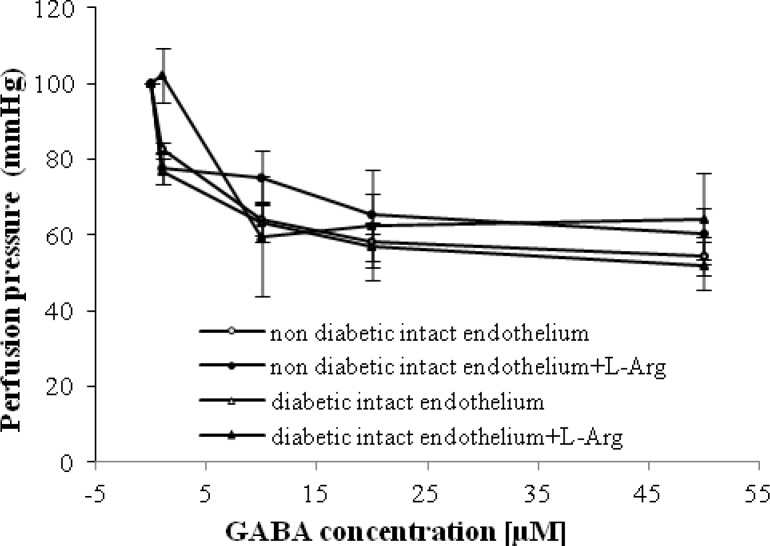
Dose response curve of gamma amino butyric acid (GABA) in mesenteric vascular beds with intact endothelium in non-diabetic and diabetic rats in presence and absence of L-Arg (six rats in each group. Data were expressed as mean ± SEM.

We observed that GABA-induced relaxation in intact mesenteric beds of non-diabetic and diabetic animals in the presence of L-Arg (0.01 M) was significantly increased at doses of 1 and 50 µM in non-diabetic and diabetic groups, respectively (Fig. 5). 

## DISCUSSION

The present study was designed to determine the role of GABA_B_ receptor and L-Arg in GABA-induced vasorelaxation in normal and STZ-induced diabetic rat vessels. As it was expected, the baseline perfusion pressure in STZ diabetic group was significantly higher than non-diabetic rats in both intact and denuded endothelium. This finding is in contrast with our previous studies [8, 13, 14]. In the present study, we showed that in the presence of faclofen, GABA-induced relaxation in intact and denuded endothelium mesenteric beds of STZ diabetic animals was suppressed and perfusion pressure did not change. Therefore, it seems that a part of GABA-induced vasorelaxation in STZ diabetic animals are mediated by the GABA_B_ receptor, which is presented in this investigation for the first time. GABA_B_ receptor is metabotropic transmembrane receptors that are linked via G-proteins to potassium channels. The increasing potassium concentrations hyperpolarize the cell at the end of an action potential [15]. In addition, as demonstrated in Figure 3, it is imaginable that the GABA_B_ receptor is placed on the smooth muscle, because no significant differences are observed in perfusion pressure with the presence of faclofen in STZ diabetic group after endothelial removal. However, according to our finding in Figure 4, perfusion pressure in STZ diabetic group was suppressed with the presence of bicuculline, and his response is not different from GABA-induced vasorelaxation in the presence of faclofen. Therefore, it seems that GABA-induced relaxation in diabetic vessel is mediated by both GABA_A_ and B receptors, but both receptors should be available for GABA vasodilatory action. We also interestingly observed a significant vasorelaxation by GABA in the presence of faclofen in non-diabetic animals with intact and denuded endothelium. Thus, this finding and our previous data [9], the responsible mechanism for GABA-induced relaxation in non-diabetic vessels is mediated via GABA_A_ receptor and nitric oxide. 

The results of the current study showed that in the presence of L-Arg, GABA-induced relaxation in intact mesenteric beds of non-diabetic and STZ diabetic rats were raised at doses of 1 and 10 µM, respectively (Fig. 5). No significant differences were observed at a GABA concentration of 1 µM in the presence of L-Arg in STZ diabetic group with intact endothelium (Fig. 5), which confirmed our previous study [9]. Therefore, it may be inferred that the low-dose GABA relaxatory effect is not mediated by the nitric oxide system in both diabetic and non-diabetic groups. However, in diabetic group, if GABA binds to the GABA_A_ or GABA_B_ receptors at doses of GABA, we need to have the normal level of nitric oxide, because in the presence of L-Arg, low-dose GABA relaxatory effect was not observed. 

Based on this investigation, we conclude that GABA-induced vasorelaxation in diabetic animals is mediated by GABAA and GABAB receptors and nitric oxide, but this response in non-diabetic group just mediated by GABAA receptor and nitric oxide. 
